# 2-(1*H*-Benzotriazol-1-yl)-1-(3-bromo­benzo­yl)ethyl benzoate

**DOI:** 10.1107/S1600536809002608

**Published:** 2009-01-28

**Authors:** Kong-Cheng Hu, Wei Wang

**Affiliations:** a College of Life Science and Pharmaceutical Engineering, Nanjing University of Technology, 210009 Nanjing, Jiangsu, People’s Republic of China

## Abstract

In the title compound, C_22_H_16_BrN_3_O_3_, the dihedral angles between the benzotriazole mean plane and the benzene rings of 4.84 (1) and 89.50 (1)°. The dihedral angle between the two benzene rings is 84.77 (1)°. In the crystal structure, mol­ecules are linked into chains by inter­molecular C—H⋯O hydrogen bonds. The crystal packing is further stabilized by C—H⋯π inter­actions.

## Related literature

For general background on benzotriazoles, see: Xu *et al.* (2003 ▶). For the synthesis, see: Zhang *et al.* (2006 ▶). For bond-length data, see: Allen *et al.* (1987 ▶).
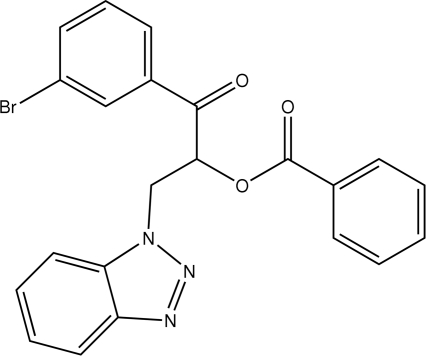


## Experimental

### 

#### Crystal data

C_22_H_16_BrN_3_O_3_
*M* *_r_* = 450.28
Triclinic, 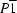

*a* = 6.4390 (12) Å
*b* = 8.8267 (17) Å
*c* = 17.430 (3) Å
α = 88.589 (3)°
β = 85.024 (3)°
γ = 83.087 (3)°
*V* = 979.6 (3) Å^3^
*Z* = 2
Mo *K*α radiation
μ = 2.13 mm^−1^
*T* = 293 (2) K
0.42 × 0.12 × 0.10 mm

#### Data collection

Siemens SMART 1000 CCD area-detector diffractometer
Absorption correction: multi-scan (*SADABS*; Sheldrick, 1996 ▶) *T*_min_ = 0.469, *T*_max_ = 0.815
5526 measured reflections
3773 independent reflections
2885 reflections with *I* > 2σ(*I*)
*R*_int_ = 0.015

#### Refinement

*R*[*F* ^2^ > 2σ(*F* ^2^)] = 0.040
*wR*(*F* ^2^) = 0.101
*S* = 1.04
3773 reflections
262 parameters
H-atom parameters constrained
Δρ_max_ = 0.45 e Å^−3^
Δρ_min_ = −0.33 e Å^−3^

### 

Data collection: *SMART* (Siemens, 1996 ▶); cell refinement: *SAINT* (Siemens, 1996 ▶); data reduction: *SAINT*; program(s) used to solve structure: *SHELXS97* (Sheldrick, 2008 ▶); program(s) used to refine structure: *SHELXL97* (Sheldrick, 2008 ▶); molecular graphics: *SHELXTL* (Sheldrick, 2008 ▶); software used to prepare material for publication: *SHELXTL*, *PARST* (Nardelli, 1995 ▶) and *PLATON* (Spek, 2003 ▶).

## Supplementary Material

Crystal structure: contains datablocks global, I. DOI: 10.1107/S1600536809002608/at2710sup1.cif

Structure factors: contains datablocks I. DOI: 10.1107/S1600536809002608/at2710Isup2.hkl

Additional supplementary materials:  crystallographic information; 3D view; checkCIF report

## Figures and Tables

**Table 1:** Hydrogen-bond geometry (Å, °) *Cg*1 is the centroid of the N1–N3/C17/C18 ring.

*D*—H⋯*A*	*D*—H	H⋯*A*	*D*⋯*A*	*D*—H⋯*A*
C16—H16*A*⋯*Cg*1^i^	0.93	2.78	3.571	144
C21—H21*A*⋯O3^ii^	0.93	2.47	3.202 (4)	136

Symmetry codes: (i) 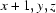; (ii) 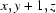.

**Fig. 1.**
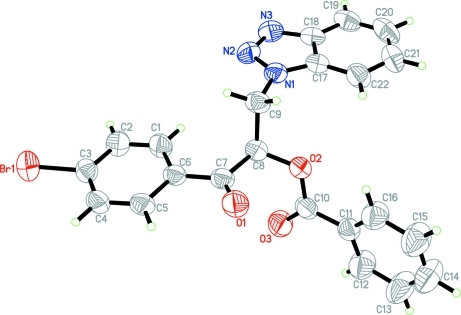
The structure of the compound (I) showing 50% probability displacement ellipsoids and the atom numbering scheme.

**Fig. 2.**
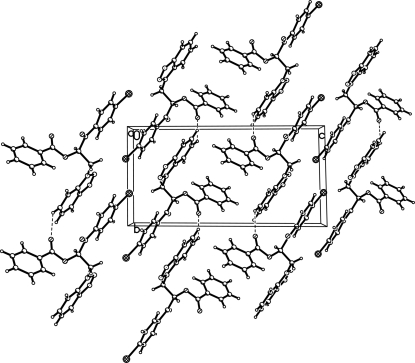
A packing diagram of (I), viewed down the a axis. Hydrogen bonds are indicated by dashed lines.

## supplementary crystallographic information

### Comment

Benzotriazole derivatives have become the most efficient antifungal compounds with the properties of low toxicity, high oral bioavailability and so on (Xu *et al.*, 2003). In order to search for new benzotriazole compounds with higher bioactivity, the title compound, (I), was synthesized, and its structure is presented here.

In the title compound (I) (Fig. 1), all bond lengths and angles are within normal ranges (Allen *et al.*, 1987). The benzotriazole ring is essentially planar with a dihedral angle of 0.29 (15)°. The whole molecule is non-planar, with the dihedral angles between the benzotriazole mean plane and C1–C6 and C11–C16 benzene rings of 4.84 (1) and 89.50 (1)°, respectively. The dihedral angle between the two benzene rings is 84.77 (1)°.

In the crystal structure (Fig. 2), molecules are linked into chains along *b* axis by C21—H21A···O3 intermolecular hydrogen bonds (Table 1). Furthermore, the crystal packing is further stabilized by C—H···π interactions (Table 1).

### Experimental

The title compound was prepared according to the literature method of Zhang *et al.* (2006). Single crystals suitable for X-ray diffraction were obtained by slow evaporation of an ethyl acetate solution at room temperature over a period of six days.

### Refinement

All H atoms were located in difference Fourier maps and constrained to ride on their parent atoms, with C—H distances in the range 0.93–0.978 Å, and with *U*_iso_(H) = 1.2 *U*_eq_(C).

### Crystal data

**Table:**

C_22_H_16_BrN_3_O_3_	*Z* = 2
*M**_r_* = 450.28	*F*(000) = 456
Triclinic, *P*1	*D*_x_ = 1.527 Mg m^−^^3^
Hall symbol: -P 1	Mo *K*α radiation, λ = 0.71073 Å
*a* = 6.4390 (12) Å	Cell parameters from 2027 reflections
*b* = 8.8267 (17) Å	θ = 2.3–24.1°
*c* = 17.430 (3) Å	µ = 2.13 mm^−^^1^
α = 88.589 (3)°	*T* = 293 K
β = 85.024 (3)°	Column, colourless
γ = 83.087 (3)°	0.42 × 0.12 × 0.10 mm
*V* = 979.6 (3) Å^3^	

### Data collection

**Table:**

Siemens SMART 1000 CCD area-detector diffractometer	3773 independent reflections
Radiation source: fine-focus sealed tube	2885 reflections with *I* > 2σ(*I*)
graphite	*R*_int_ = 0.015
Detector resolution: 8.33 pixels mm^-1^	θ_max_ = 26.0°, θ_min_ = 2.3°
ω scans	*h* = −5→7
Absorption correction: multi-scan (*SADABS*; Sheldrick, 1996)	*k* = −8→10
*T*_min_ = 0.469, *T*_max_ = 0.815	*l* = −21→21
5526 measured reflections	

### Refinement

**Table:**

Refinement on *F*^2^	Primary atom site location: structure-invariant direct methods
Least-squares matrix: full	Secondary atom site location: difference Fourier map
*R*[*F*^2^ > 2σ(*F*^2^)] = 0.040	Hydrogen site location: inferred from neighbouring sites
*wR*(*F*^2^) = 0.101	H-atom parameters constrained
*S* = 1.04	*w* = 1/[σ^2^(*F*_o_^2^) + (0.0529*P*)^2^ + 0.1162*P*] where *P* = (*F*_o_^2^ + 2*F*_c_^2^)/3
3773 reflections	(Δ/σ)_max_ < 0.001
262 parameters	Δρ_max_ = 0.45 e Å^−^^3^
0 restraints	Δρ_min_ = −0.33 e Å^−^^3^

### Special details

**Table:**

Geometry. All e.s.d.'s (except the e.s.d. in the dihedral angle between two l.s. planes) are estimated using the full covariance matrix. The cell e.s.d.'s are taken into account individually in the estimation of e.s.d.'s in distances, angles and torsion angles; correlations between e.s.d.'s in cell parameters are only used when they are defined by crystal symmetry. An approximate (isotropic) treatment of cell e.s.d.'s is used for estimating e.s.d.'s involving l.s. planes.
Refinement. Refinement of *F*^2^ against ALL reflections. The weighted *R*-factor *wR* and goodness of fit *S* are based on *F*^2^, conventional *R*-factors *R* are based on *F*, with *F* set to zero for negative *F*^2^. The threshold expression of *F*^2^ > σ(*F*^2^) is used only for calculating *R*-factors(gt) *etc*. and is not relevant to the choice of reflections for refinement. *R*-factors based on *F*^2^ are statistically about twice as large as those based on *F*, and *R*- factors based on ALL data will be even larger.

### Fractional atomic coordinates and isotropic or equivalent isotropic displacement parameters (Å^2^)

**Table:**

	*x*	*y*	*z*	*U*_iso_*/*U*_eq_	
Br1	0.15134 (5)	0.67410 (4)	0.013609 (18)	0.07266 (15)	
O1	0.8295 (3)	1.1006 (2)	−0.19239 (12)	0.0665 (5)	
O2	0.6637 (3)	1.31723 (18)	−0.28399 (10)	0.0539 (5)	
N2	0.0957 (4)	1.4833 (3)	−0.16528 (13)	0.0589 (6)	
N1	0.2970 (3)	1.4981 (2)	−0.19112 (12)	0.0493 (5)	
C3	0.2999 (4)	0.8086 (3)	−0.04996 (15)	0.0516 (6)	
O3	0.6428 (4)	1.1121 (2)	−0.35433 (12)	0.0761 (6)	
C17	0.3044 (4)	1.6208 (3)	−0.23960 (14)	0.0471 (6)	
C7	0.6396 (4)	1.1190 (3)	−0.18590 (15)	0.0490 (6)	
C4	0.5140 (4)	0.7794 (3)	−0.06486 (15)	0.0557 (7)	
H4A	0.5862	0.6915	−0.0451	0.067*	
C6	0.5140 (4)	1.0130 (3)	−0.13989 (14)	0.0471 (6)	
C8	0.5243 (4)	1.2583 (3)	−0.22507 (15)	0.0499 (6)	
H8A	0.3993	1.2305	−0.2470	0.060*	
C2	0.1912 (4)	0.9376 (3)	−0.08071 (16)	0.0582 (7)	
H2B	0.0461	0.9562	−0.0710	0.070*	
C10	0.7238 (4)	1.2242 (3)	−0.34460 (15)	0.0544 (7)	
C5	0.6206 (4)	0.8806 (3)	−0.10897 (15)	0.0535 (6)	
H5A	0.7656	0.8612	−0.1185	0.064*	
C18	0.0959 (4)	1.6813 (3)	−0.24132 (15)	0.0511 (6)	
N3	−0.0275 (4)	1.5931 (3)	−0.19522 (14)	0.0624 (6)	
C1	0.2975 (4)	1.0380 (3)	−0.12556 (16)	0.0560 (7)	
H1A	0.2235	1.1238	−0.1466	0.067*	
C9	0.4638 (4)	1.3866 (3)	−0.16730 (15)	0.0541 (6)	
H9A	0.4194	1.3430	−0.1178	0.065*	
H9B	0.5863	1.4376	−0.1608	0.065*	
C22	0.4686 (5)	1.6847 (3)	−0.28088 (18)	0.0610 (7)	
H22A	0.6080	1.6432	−0.2793	0.073*	
C11	0.9024 (4)	1.2743 (3)	−0.39390 (15)	0.0535 (6)	
C21	0.4115 (5)	1.8121 (3)	−0.3239 (2)	0.0726 (9)	
H21A	0.5153	1.8587	−0.3526	0.087*	
C19	0.0423 (5)	1.8118 (3)	−0.28590 (18)	0.0631 (8)	
H19A	−0.0968	1.8536	−0.2879	0.076*	
C12	0.9535 (6)	1.2079 (4)	−0.46490 (18)	0.0778 (9)	
H12A	0.8719	1.1376	−0.4820	0.093*	
C16	1.0265 (5)	1.3774 (4)	−0.36929 (18)	0.0721 (9)	
H16A	0.9933	1.4227	−0.3215	0.086*	
C20	0.2004 (6)	1.8749 (3)	−0.32606 (19)	0.0748 (9)	
H20A	0.1688	1.9621	−0.3559	0.090*	
C14	1.2476 (6)	1.3470 (5)	−0.4857 (2)	0.0931 (11)	
H14A	1.3641	1.3710	−0.5169	0.112*	
C15	1.1998 (6)	1.4135 (5)	−0.4154 (2)	0.0915 (11)	
H15A	1.2835	1.4827	−0.3986	0.110*	
C13	1.1247 (6)	1.2457 (5)	−0.5102 (2)	0.0958 (12)	
H13A	1.1575	1.2017	−0.5583	0.115*	

### Atomic displacement parameters (Å^2^)

**Table:**

	*U*^11^	*U*^22^	*U*^33^	*U*^12^	*U*^13^	*U*^23^
Br1	0.0764 (3)	0.0699 (2)	0.0733 (2)	−0.01787 (16)	−0.00824 (16)	0.01757 (16)
O1	0.0458 (12)	0.0633 (12)	0.0861 (14)	0.0045 (9)	−0.0008 (10)	0.0130 (10)
O2	0.0662 (12)	0.0404 (9)	0.0529 (11)	−0.0038 (8)	0.0035 (9)	−0.0007 (8)
N2	0.0512 (14)	0.0594 (14)	0.0609 (14)	0.0033 (11)	0.0100 (11)	0.0006 (11)
N1	0.0467 (12)	0.0456 (12)	0.0524 (12)	0.0060 (9)	−0.0012 (10)	−0.0012 (9)
C3	0.0587 (17)	0.0502 (15)	0.0470 (14)	−0.0080 (12)	−0.0096 (12)	0.0009 (11)
O3	0.1015 (17)	0.0571 (12)	0.0724 (14)	−0.0305 (12)	0.0110 (12)	−0.0155 (10)
C17	0.0480 (15)	0.0415 (13)	0.0508 (14)	0.0003 (11)	−0.0052 (11)	−0.0084 (11)
C7	0.0484 (16)	0.0452 (14)	0.0505 (15)	0.0058 (11)	−0.0038 (12)	−0.0022 (11)
C4	0.0607 (18)	0.0470 (15)	0.0564 (16)	0.0070 (12)	−0.0089 (13)	0.0052 (12)
C6	0.0468 (15)	0.0446 (14)	0.0477 (14)	0.0042 (11)	−0.0048 (11)	−0.0015 (11)
C8	0.0499 (15)	0.0448 (14)	0.0533 (15)	0.0007 (11)	−0.0031 (12)	0.0011 (11)
C2	0.0429 (15)	0.0668 (18)	0.0640 (17)	−0.0027 (13)	−0.0073 (13)	0.0078 (14)
C10	0.0697 (18)	0.0393 (14)	0.0529 (16)	−0.0020 (13)	−0.0046 (13)	−0.0002 (12)
C5	0.0464 (15)	0.0509 (15)	0.0597 (16)	0.0084 (12)	−0.0055 (12)	0.0009 (12)
C18	0.0504 (16)	0.0462 (14)	0.0551 (16)	0.0057 (12)	−0.0070 (12)	−0.0133 (12)
N3	0.0502 (14)	0.0616 (15)	0.0707 (16)	0.0059 (11)	0.0052 (12)	−0.0054 (12)
C1	0.0476 (16)	0.0549 (16)	0.0633 (17)	0.0046 (12)	−0.0095 (13)	0.0093 (13)
C9	0.0585 (16)	0.0489 (15)	0.0519 (15)	0.0098 (12)	−0.0093 (12)	−0.0006 (12)
C22	0.0541 (17)	0.0493 (16)	0.080 (2)	−0.0081 (13)	−0.0049 (14)	−0.0004 (14)
C11	0.0685 (18)	0.0447 (14)	0.0460 (14)	−0.0036 (12)	−0.0016 (12)	0.0014 (11)
C21	0.084 (2)	0.0511 (17)	0.085 (2)	−0.0206 (16)	−0.0047 (18)	0.0065 (15)
C19	0.0685 (19)	0.0480 (16)	0.0713 (19)	0.0103 (14)	−0.0190 (15)	−0.0072 (14)
C12	0.095 (2)	0.083 (2)	0.0592 (19)	−0.0282 (19)	0.0034 (17)	−0.0146 (17)
C16	0.083 (2)	0.075 (2)	0.0601 (18)	−0.0211 (17)	0.0040 (16)	−0.0129 (15)
C20	0.103 (3)	0.0417 (16)	0.081 (2)	0.0013 (16)	−0.0285 (19)	0.0060 (15)
C14	0.088 (3)	0.121 (3)	0.072 (2)	−0.034 (2)	0.017 (2)	−0.006 (2)
C15	0.093 (3)	0.105 (3)	0.082 (2)	−0.043 (2)	0.009 (2)	−0.017 (2)
C13	0.109 (3)	0.120 (3)	0.059 (2)	−0.035 (3)	0.021 (2)	−0.020 (2)

### Geometric parameters (Å, °)

**Table:**

Br1—C3	1.891 (3)	C5—H5A	0.9300
O1—C7	1.210 (3)	C18—N3	1.375 (4)
O2—C10	1.355 (3)	C18—C19	1.399 (4)
O2—C8	1.435 (3)	C1—H1A	0.9300
N2—N3	1.305 (3)	C9—H9A	0.9700
N2—N1	1.355 (3)	C9—H9B	0.9700
N1—C17	1.360 (3)	C22—C21	1.367 (4)
N1—C9	1.448 (3)	C22—H22A	0.9300
C3—C4	1.374 (4)	C11—C12	1.379 (4)
C3—C2	1.385 (4)	C11—C16	1.380 (4)
O3—C10	1.195 (3)	C21—C20	1.409 (5)
C17—C18	1.386 (4)	C21—H21A	0.9300
C17—C22	1.394 (4)	C19—C20	1.353 (4)
C7—C6	1.485 (4)	C19—H19A	0.9300
C7—C8	1.535 (3)	C12—C13	1.369 (5)
C4—C5	1.372 (4)	C12—H12A	0.9300
C4—H4A	0.9300	C16—C15	1.382 (4)
C6—C1	1.387 (4)	C16—H16A	0.9300
C6—C5	1.402 (3)	C20—H20A	0.9300
C8—C9	1.527 (4)	C14—C13	1.362 (5)
C8—H8A	0.9800	C14—C15	1.368 (5)
C2—C1	1.373 (4)	C14—H14A	0.9300
C2—H2B	0.9300	C15—H15A	0.9300
C10—C11	1.480 (4)	C13—H13A	0.9300
			
C10—O2—C8	115.31 (19)	C2—C1—C6	120.8 (2)
N3—N2—N1	108.8 (2)	C2—C1—H1A	119.6
N2—N1—C17	110.4 (2)	C6—C1—H1A	119.6
N2—N1—C9	119.2 (2)	N1—C9—C8	112.8 (2)
C17—N1—C9	130.4 (2)	N1—C9—H9A	109.0
C4—C3—C2	120.3 (2)	C8—C9—H9A	109.0
C4—C3—Br1	120.3 (2)	N1—C9—H9B	109.0
C2—C3—Br1	119.3 (2)	C8—C9—H9B	109.0
N1—C17—C18	103.9 (2)	H9A—C9—H9B	107.8
N1—C17—C22	133.2 (2)	C21—C22—C17	115.7 (3)
C18—C17—C22	122.9 (2)	C21—C22—H22A	122.1
O1—C7—C6	122.2 (2)	C17—C22—H22A	122.1
O1—C7—C8	119.0 (2)	C12—C11—C16	119.2 (3)
C6—C7—C8	118.8 (2)	C12—C11—C10	118.4 (3)
C5—C4—C3	119.7 (2)	C16—C11—C10	122.3 (2)
C5—C4—H4A	120.1	C22—C21—C20	122.1 (3)
C3—C4—H4A	120.1	C22—C21—H21A	119.0
C1—C6—C5	118.3 (2)	C20—C21—H21A	119.0
C1—C6—C7	123.6 (2)	C20—C19—C18	117.5 (3)
C5—C6—C7	118.1 (2)	C20—C19—H19A	121.2
O2—C8—C9	105.9 (2)	C18—C19—H19A	121.2
O2—C8—C7	109.4 (2)	C13—C12—C11	119.9 (3)
C9—C8—C7	110.1 (2)	C13—C12—H12A	120.1
O2—C8—H8A	110.4	C11—C12—H12A	120.1
C9—C8—H8A	110.4	C11—C16—C15	120.3 (3)
C7—C8—H8A	110.4	C11—C16—H16A	119.9
C1—C2—C3	120.0 (2)	C15—C16—H16A	119.9
C1—C2—H2B	120.0	C19—C20—C21	121.7 (3)
C3—C2—H2B	120.0	C19—C20—H20A	119.1
O3—C10—O2	122.4 (2)	C21—C20—H20A	119.1
O3—C10—C11	125.0 (2)	C13—C14—C15	120.0 (3)
O2—C10—C11	112.5 (2)	C13—C14—H14A	120.0
C4—C5—C6	121.0 (2)	C15—C14—H14A	120.0
C4—C5—H5A	119.5	C14—C15—C16	119.7 (3)
C6—C5—H5A	119.5	C14—C15—H15A	120.1
N3—C18—C17	109.1 (2)	C16—C15—H15A	120.1
N3—C18—C19	130.8 (3)	C14—C13—C12	120.9 (3)
C17—C18—C19	120.1 (3)	C14—C13—H13A	119.5
N2—N3—C18	107.9 (2)	C12—C13—H13A	119.5
			
N3—N2—N1—C17	−0.3 (3)	N1—N2—N3—C18	−0.2 (3)
N3—N2—N1—C9	−178.5 (2)	C17—C18—N3—N2	0.5 (3)
N2—N1—C17—C18	0.6 (3)	C19—C18—N3—N2	−179.8 (3)
C9—N1—C17—C18	178.5 (2)	C3—C2—C1—C6	−0.8 (4)
N2—N1—C17—C22	−180.0 (3)	C5—C6—C1—C2	1.6 (4)
C9—N1—C17—C22	−2.0 (5)	C7—C6—C1—C2	−178.1 (3)
C2—C3—C4—C5	1.5 (4)	N2—N1—C9—C8	94.5 (3)
Br1—C3—C4—C5	−177.8 (2)	C17—N1—C9—C8	−83.3 (3)
O1—C7—C6—C1	174.0 (3)	O2—C8—C9—N1	81.7 (3)
C8—C7—C6—C1	−4.7 (4)	C7—C8—C9—N1	−160.1 (2)
O1—C7—C6—C5	−5.7 (4)	N1—C17—C22—C21	−179.5 (3)
C8—C7—C6—C5	175.6 (2)	C18—C17—C22—C21	−0.1 (4)
C10—O2—C8—C9	−175.1 (2)	O3—C10—C11—C12	14.4 (4)
C10—O2—C8—C7	66.3 (3)	O2—C10—C11—C12	−167.9 (3)
O1—C7—C8—O2	18.8 (3)	O3—C10—C11—C16	−161.4 (3)
C6—C7—C8—O2	−162.4 (2)	O2—C10—C11—C16	16.3 (4)
O1—C7—C8—C9	−97.1 (3)	C17—C22—C21—C20	0.2 (5)
C6—C7—C8—C9	81.6 (3)	N3—C18—C19—C20	−179.9 (3)
C4—C3—C2—C1	−0.8 (4)	C17—C18—C19—C20	−0.3 (4)
Br1—C3—C2—C1	178.6 (2)	C16—C11—C12—C13	−0.5 (5)
C8—O2—C10—O3	10.9 (4)	C10—C11—C12—C13	−176.5 (3)
C8—O2—C10—C11	−166.9 (2)	C12—C11—C16—C15	0.0 (5)
C3—C4—C5—C6	−0.7 (4)	C10—C11—C16—C15	175.8 (3)
C1—C6—C5—C4	−0.8 (4)	C18—C19—C20—C21	0.4 (5)
C7—C6—C5—C4	178.9 (2)	C22—C21—C20—C19	−0.4 (5)
N1—C17—C18—N3	−0.7 (3)	C13—C14—C15—C16	0.0 (7)
C22—C17—C18—N3	179.8 (2)	C11—C16—C15—C14	0.3 (6)
N1—C17—C18—C19	179.6 (2)	C15—C14—C13—C12	−0.5 (7)
C22—C17—C18—C19	0.1 (4)	C11—C12—C13—C14	0.8 (6)

### Hydrogen-bond geometry (Å, °)

**Table:**

*D*—H···*A*	*D*—H	H···*A*	*D*···*A*	*D*—H···*A*
C16—H16A···Cg1^i^	0.93	2.78	3.571	144
C21—H21A···O3^ii^	0.93	2.47	3.202 (4)	136

Symmetry codes: (i) *x*+1, *y*, *z*; (ii) *x*, *y*+1, *z*.

## References

[bb1] Allen, F. H., Kennard, O., Watson, D. G., Brammer, L., Orpen, A. G. & Taylor, R. (1987). *J. Chem. Soc. Perkin Trans. 2*, pp. S1–19.

[bb2] Nardelli, M. (1995). *J. Appl. Cryst.* **28**, 659.

[bb3] Sheldrick, G. M. (1996). *SADABS* University of Göttingen, Germany.

[bb4] Sheldrick, G. M. (2008). *Acta Cryst.* A**64**, 112–122. 10.1107/S0108767307043930 18156677

[bb5] Siemens (1996). *SMART* and *SAINT* Siemens Analytical X-ray Instruments Inc., Madison, Wisconsin, USA.

[bb6] Spek, A. L. (2003). *J. Appl. Cryst.* **36**, 7–13.

[bb7] Xu, L. Z., Zhang, S. S. & Hu, Z. Q. (2003). *Chem. Res. Chin. Univ.* **19**, 310–313.

[bb8] Zhang, S.-S., Wan, J., Peng, Z.-Z. & Bi, S. (2006). *Acta Cryst.* E**62**, o4348–o4349.

