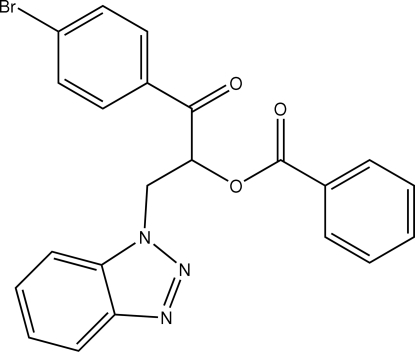# 2-(1*H*-Benzotriazol-1-yl)-1-(3-bromo­benzo­yl)ethyl benzoate. Corrigendum

**DOI:** 10.1107/S1600536809029390

**Published:** 2009-07-31

**Authors:** Kong-Cheng Hu, Wei Wang

**Affiliations:** a College of Life Science and Pharmaceutical Engineering, Nanjing University of Technology, 210009 Nanjing, Jiangsu, People’s Republic of China

## Abstract

The chemical name of the title compound in the paper by Hu & Wang [*Acta Cryst.* (2009), E**65**, o392] is corrected and the structural diagram is updated.

## Abstract 2

Corrigendum to *Acta Cryst.* (2009), E**65**, o392.

In the paper by Hu & Wang [*Acta Cryst.* (2009), E**65**, o392], the chemical name given in the *Title* should be ‘2-(1*H*-Benzotriazol-1-yl)-1-(4-bromo­benzo­yl)ethyl benzoate’. An updated structural diagram is shown below.